# Pharmacological Inhibition of Sonic Hedgehog Signaling Suppresses Tumor Development in a Murine Model of Intrahepatic Cholangiocarcinoma

**DOI:** 10.3390/ijms222413214

**Published:** 2021-12-08

**Authors:** Kyungjoo Cho, Hyuk Moon, Sang Hyun Seo, Simon Weonsang Ro, Beom Kyung Kim

**Affiliations:** 1 Brain Korea 21 Plus Project for Medical Science College of Medicine, Yonsei University, Seoul 03722, Korea; kyungjoo89@yuhs.ac (K.C.); sshing94@yuhs.ac (S.H.S.); 2 Department of Genetics and Biotechnology, College of Life Sciences, Kyung Hee University, Yongin 17104, Korea; hmoon@khu.ac.kr; 3 Department of Internal Medicine, Yonsei University College of Medicine, Seoul 03722, Korea; 4 Institute of Gastroenterology, Yonsei University College of Medicine, Seoul 03722, Korea

**Keywords:** cholangiocarcinoma, hydrodynamic transfection, sonic hedgehog, hedgehog pathway inhibitor, molecular target therapy

## Abstract

Cholangiocarcinoma (CCC) is the second most primary liver cancer with an aggressive biological behavior, and its incidence increases steadily. An aberrant up-regulation of the sonic hedgehog signaling pathway has been reported in a variety of hepatic diseases including hepatic inflammation, fibrosis, as well as cancer. In this study, we determined the effect of a sonic hedgehog inhibitor, vismodegib, on the development of CCC. Through database analyses, we found sonic hedgehog signaling was up-regulated in human CCC, based on overexpression of its target genes, *GLI1* and *GLI2*. Further, human CCC cells were highly sensitive to the treatment with vismodegib in vitro. Based on the data, we investigated the in vivo anti-cancer efficacy of vismodegib in CCC employing a murine model of CCC developed by hydrodynamic tail vein injection method. In the murine model, CCC induced by constitutively active forms of TAZ and PI3K exhibited up-regulated sonic hedgehog signaling. Treatment of vismodegib significantly suppressed tumor development in the murine CCC model, based on comparison of gross morphologies and liver weight/body weight. It is expected that pharmacological inhibition of sonic hedgehog signaling would be an effective molecular target therapy for CCC.

## 1. Introduction

Cholangiocarcinoma (CCC) is the 2nd most primary liver cancer with an aggressive biological behavior and accounts for about 15% of cases, following hepatocellular carcinoma [1]. It is a highly heterogeneous disease entity arising from neoplastic transformation at both intra- and extra-hepatic biliary epithelial cells. The risk of developing CCC has been strongly associated with chronic inflammation of the biliary tract, resulting from a heterogeneous group of several risk factors. The molecular mechanisms underlying their pathogenesis are still poorly understood. 

Among various kinds of mechanisms associated with developing CCC [2,3], an aberrant up-regulation of the hedgehog (HH) signaling pathway has been demonstrated to be involved in both initiation and progression of carcinogenesis, which is also involved in other hepato-biliary tumors such as hepatocellular carcinoma, hepatoblastoma, and gallbladder cancer. In the absence of sonic HH (SHH) which is a secreted glycoprotein member of the HH family [4], the 12-transmembrane receptor Patched 1 (PTCH1) normally inhibits G-coupled 7-transmembrane protein Smoothened (SMO). Upon binding of SHH to Patched 1, the receptor-mediated suppression of SMO is abolished, and SMO now can activate the downstream signaling pathway, leading to translocation of GLI family transcription factors to the nucleus. Numerous SHH target genes are transcribed in this manner that include *GLI1* and *GLI2* in a positive feedback loop [3]. Genetic alteration in both mutation and copy number of *GLI1* and *GLI2* had been observed in CCC [5,6]. 

Vismodegib (GDC-0449) is the 2nd-generation cyclopamine derivative and the first oral systemic HH pathway inhibitor (HPI) approved by the Food and Drug Administration (FDA) and European Medicines Agency (EMA) [7]. It binds to the SMO receptor, thereby inhibiting its action and inhibiting tumor growth. Currently, it is the first-line anti-cancer medication for patients with locally advanced basal cell carcinomas or metastatic basal cell carcinomas where radiotherapy and surgery are not eligible [8].

Given that the SHH signaling pathway is up-regulated in about 50% of CCC [9], we aimed to assess whether CCC with up-regulated SHH signaling is also vulnerable to HPI. For this purpose, we employed the hydrodynamics-based transfection method to develop a murine autochthonous intrahepatic CCC model, and then investigated the effects of HPI on tumor development.

## 2. Results

### 2.1. Activation of SHH Signaling in Human Intrahepatic CCC

**Figure 1:**
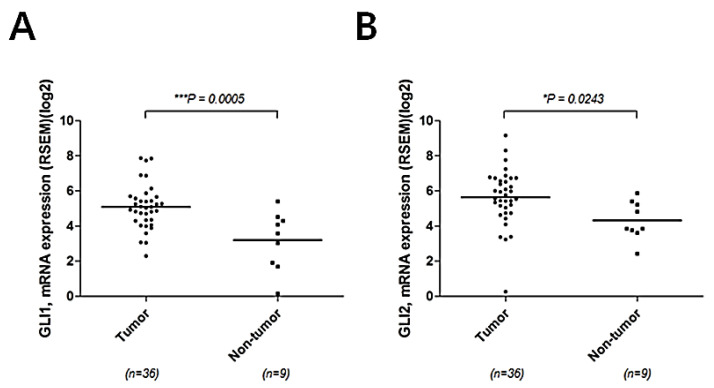
Activation of SHH signaling in human CCC. Expression levels of GLI1 (**A**) and GLI2 (**B**) in tumor and non-tumor tissues were compared using The Cancer Genome Atlas (TCGA).

First, we verified that SHH activity was elevated in human intrahepatic CCC. Elevated expression of *GLI1* and *GLI2* is commonly used as a marker for SHH [10,11]. Using publicly available databases, The Cancer Genome Atlas (TCGA), we compared expression levels of *GLI1* and *GLI2* in intrahepatic CCC with those in non-tumor liver tissues. Significant overexpression of both *GLI1* and *GLI2* was found in intrahepatic CCC, when compared with non-tumor tissues (*p* < 0.001 and 0.05, respectively), suggesting a possible tumorigenic effect of SHH signaling during the development of CCC (Figure 1A,B). 

### 2.2. Sensitivity of Human Intrahepatic CCC Cells to a Chemical Inhibitor of SHH Signaling

**Figure 2:**
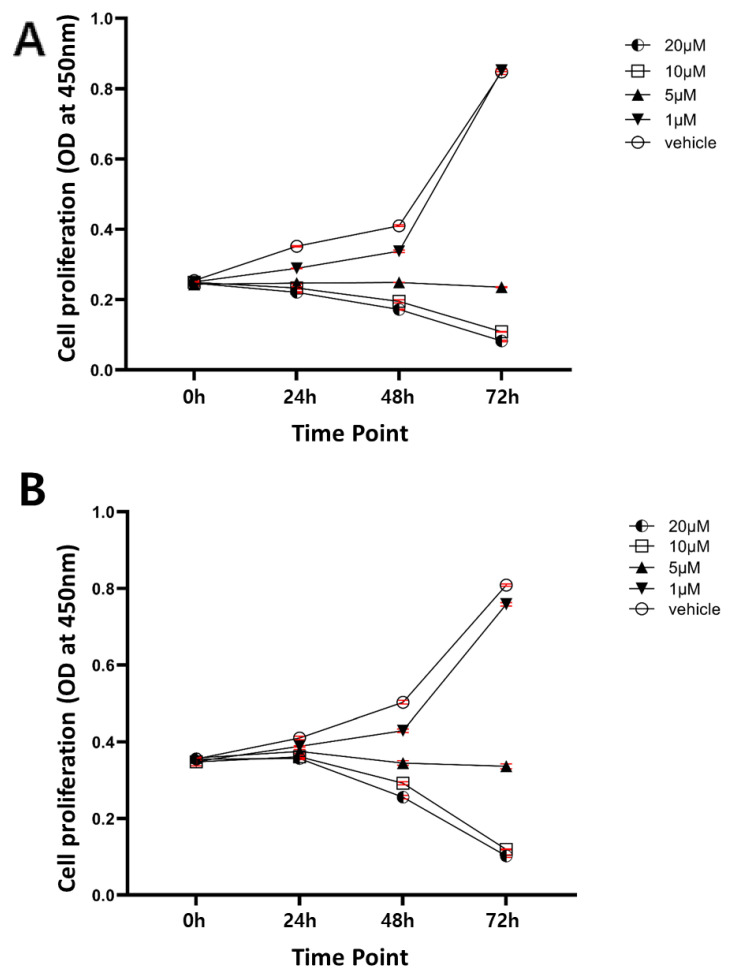
Vismodegib inhibits proliferation of CCC cells. The numbers of live cells were estimated using an MTT-based assay kit at the indicated time points after treating CCC cells with vismodegib at 0, 1, 5, 10, and 20 μM. (**A**) SNU-1079; (**B**) SNU-245. Data are presented as the mean ± standard error of mean (SEM).

**Figure 3:**
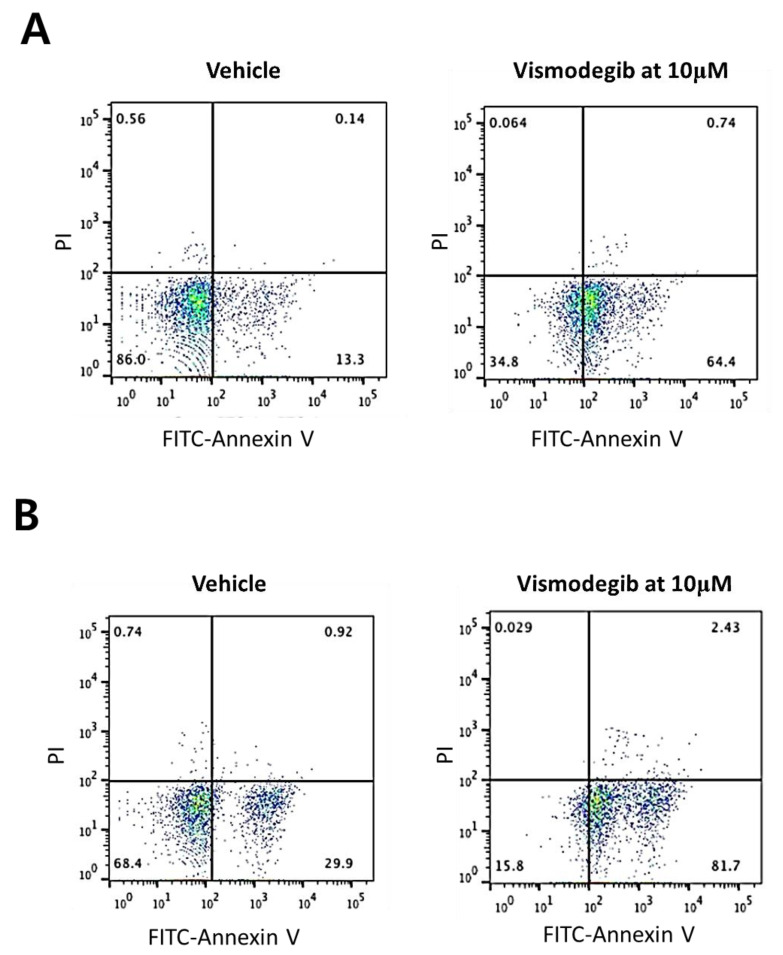
Treatment with vismodegib at 10 μM induced apoptosis in CCC cells. The y-axis represents PI staining and x-axis represents Annexin V staining. (**A**) SNU-1079; (**B**) SNU-245.

Next, we investigated sensitivities of human intrahepatic CCC cells to a chemical inhibitor of SHH, vismodegib, an effective SHH pathway inhibitor approved by the FDA. Two human CCC cell lines, SNU-1079 and SNU-245, were used for the study. Treatment with vismodegib showed a dose-dependent inhibition of cell proliferation in both cell lines. Vismodegib treatment at a dose of 5 μM halted proliferation of CCC cells, and at the concentration of 10 μM and higher, the effects were more dramatic leading to decreases in cell numbers in both cell lines (Figure 2A,B). To investigate the nature of cell deaths after the treatment with vismodegib at 10 μM, we performed Propidium Iodide (PI) and Fluorescein (FITC)-Annexin V staining and analyzed stained cells by fluorescence-activated cell sorting (FACS). The FACS experiment revealed that the treatment of CCC cells with vismodegib at 10 μM led to apoptotic cell deaths in both CCC cell lines (Figure 3A,B). The data show that human CCC cells are sensitive to HH pathway inhibitor (HPI) in vitro and proposes that HPI could be utilized as an effective target therapeutic for CCC.

### 2.3. Activation of SHH Signaling in a Murine Model of CCC Induced by Activated Forms of TAZ and PI3K

**Figure 4:**
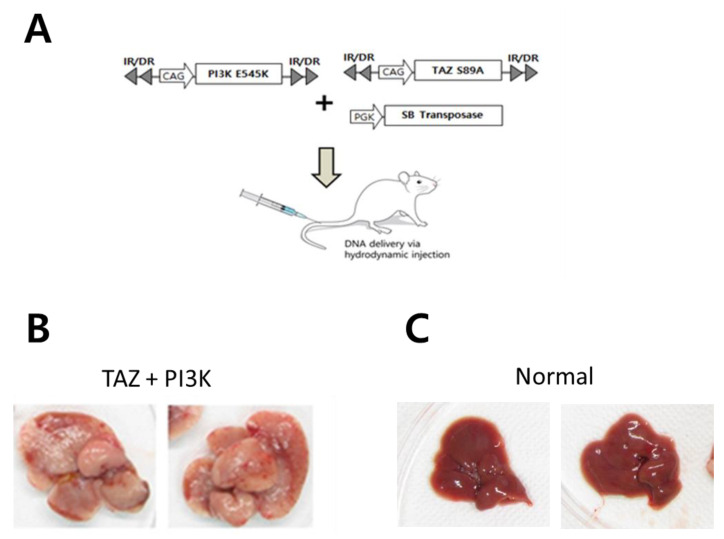
PI3K^E545K^ and TAZ^S89A^ induces intrahepatic CCC. (**A**) Schematic illustration of hydrodynamic tail vein injection (HTVI). (**B**) The gross morphology of representative livers expressing PI3K^E545K^ and TAZ^S89A^. Livers were harvested at 6 weeks following HTVI. (**C**) The gross morphology of normal livers.

To investigate whether HPI affects tumor development of CCC in the liver, we employed a simple liver-specific transgenic approach in which transposons encoding constitutively active forms of TAZ (TAZ^S89A^) and PI3K (PI3KCA^E545K^) were co-delivered to murine livers through hydrodynamic tail vein injection (HTVI) (Figure 4A). Previously, it has been reported that similar combination of oncogenes (i.e., Yap^S127A^ and PIK3CA^H1047R^) could induce CCC in murine livers [12]. At 5 to 6 weeks after HTVI, mice started to display discomfort and livers harvested from the mice showed very enlarged shapes compared with normal livers (Figure 4B,C). Numerous nodules were observed from all livers expressing TAZ^S89A^ and PI3KCA^E545K^, demonstrating high tumorigenicity by the combination of oncogenes. 

**Figure 5:**
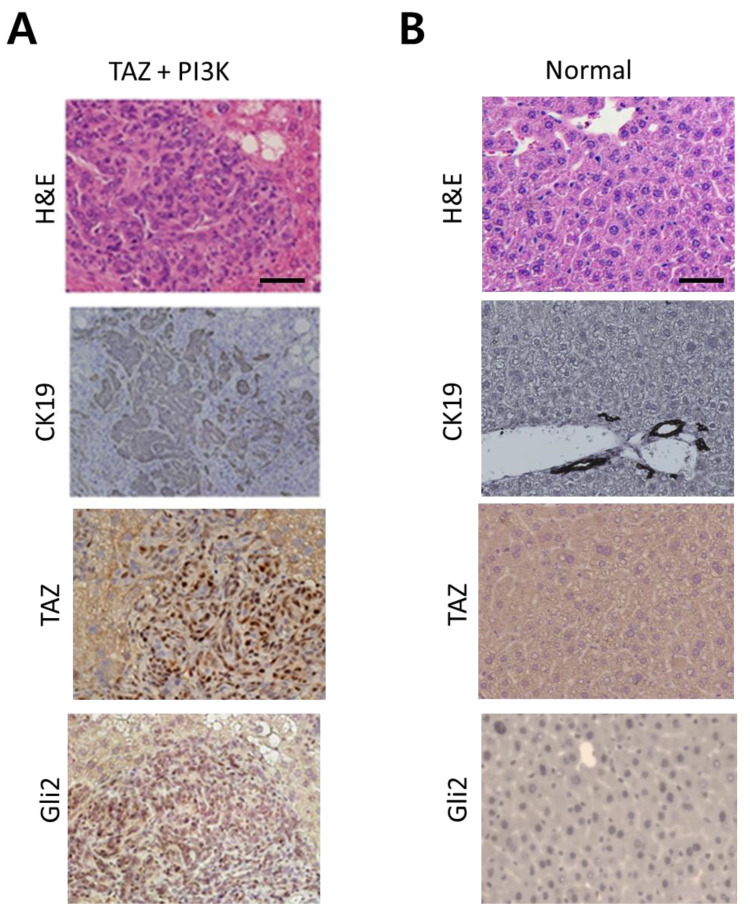
Microscopic analysis of intrahepatic CCC induced by PI3K^E545K^ and TAZ^S89A^ (**A**) H&E and IHC staining of tumor sections from the livers shown in Figure 4B. (**B**) H&E and IHC staining of sections from normal livers. Note that only bile ducts cells in normal livers were stained positive for CK19, while intrahepatic CCC showed ubiquitous expression of CK19. Scale bar, 50 μm.

Microscopic examination revealed that the tumors induced by TAZ^S89A^ and PI3KCA^E545K^ were intrahepatic CCC with high expression of CK19, a biliary cell marker (Figure 5A). The staining result of CCC tumors showed striking differences from that of normal liver tissues where only cells of bile ducts are stained positive for CK19 (Figure 5B). Of note, nuclei in neoplastic cells in CCC tumors were stained positive for Gli2, a marker for SHH activation (Figure 5A). Thus, our murine model of intrahepatic CCC induced by TAZ^S89A^ and PI3KCA^E545K^ also revealed a high activity of SHH in CCC tumors.

### 2.4. Vismodegib Suppresses Tumor Development in the Murine Model of CCC

**Figure 6:**
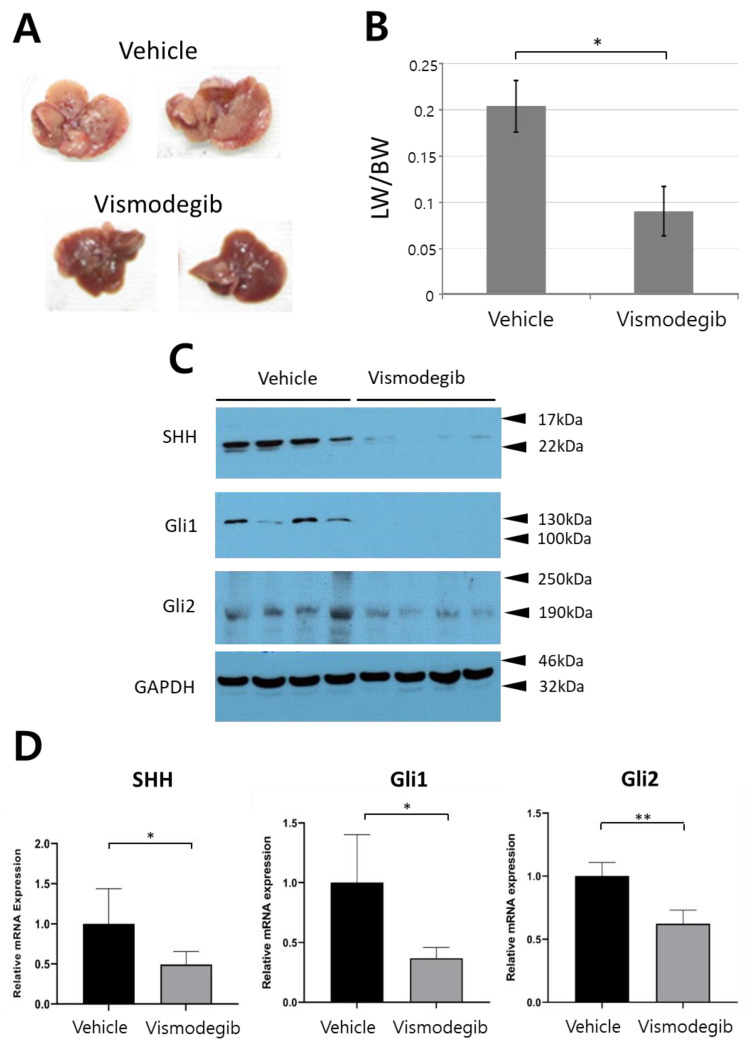
Vismodegib treatment suppressed CCC. (**A**) Gross morphology of representative livers of mice treated with vehicle and vismodegib. (**B**) Liver weight/body weight (LW/BW) ratios of mice treated with vehicle and vismodegib. The graph represents the mean ± SEM (*n* = 5 livers per group) (*, *p* < 0.05). Western blots (**C**) and quantitative RT-PCR (**D**) showing expression levels of SHH, Gli1, and Gli2 in livers of indicated groups (*, *p* < 0.05; **, *p* < 0.01).

Considering that our murine model of intrahepatic CCC well mimics human CCC, which is characterized by a high activity of SHH, we utilized the murine model to test the in vivo efficacy of HPI, vismodegib. Mice of the treated group were administered vismodegib at a daily dose of 50 mg/kg given intraperitoneally, while control mice were given vehicle (10% DMSO). After 4-week administration, livers were harvested from both the treated and control groups. Numbers of nodules and sizes of livers were significantly reduced in mice treated with vismodegib (Figure 6A). Liver weight per body weight (LW/BW), often used to evaluate tumor burden in liver, was also significantly reduced in the treated group compared with the control (Figure 6B).

To confirm downregulation of SHH signaling due to the treatment with vismodegib, we assessed the protein levels of SHH, the ligand that triggers the downstream SHH signaling pathway (Figure 6C). Additionally, protein levels of representative SHH target genes, Gli1, and Gli2 were assessed in tumors treated with vismodegib and vehicle. The protein levels of SHH, Gli1, and Gli2 were all significantly reduced in mice treated with vismodegib, compared with those in control mice (Figure 6C). Quantitative reverse-transcription PCR (RT-PCR) also confirmed decreased expression of SHH, Gli1, and Gli2 in vismodegib-treated mice (Figure 6D).

## 3. Discussion

A plethora of SHH target genes are involved in various aspects of carcinogenesis, including cell cycle progression, apoptosis, epithelial-to-mesenchymal transition, and angiogenesis [13]. Aberrant activation of Hedgehog signaling has been reported in livers with various pathological conditions such as inflammation, liver regeneration, vascular remodeling, fibrosis, and cancer [14,15,16].

In this study, we investigated the role of SHH signaling in intrahepatic CCC by employing human intrahepatic CCC cells in vitro (Figure 2 and Figure 3) as well as a murine model in vivo (Figure 4, Figure 5 and Figure 6). Pharmacological suppression of the SHH signaling pathway led to dramatic inhibition of cell proliferation in vitro and tumor growth in vivo, proposing that HPI can be an effective and attractive molecular target therapy for human intrahepatic CCC.

**Figure 7:**
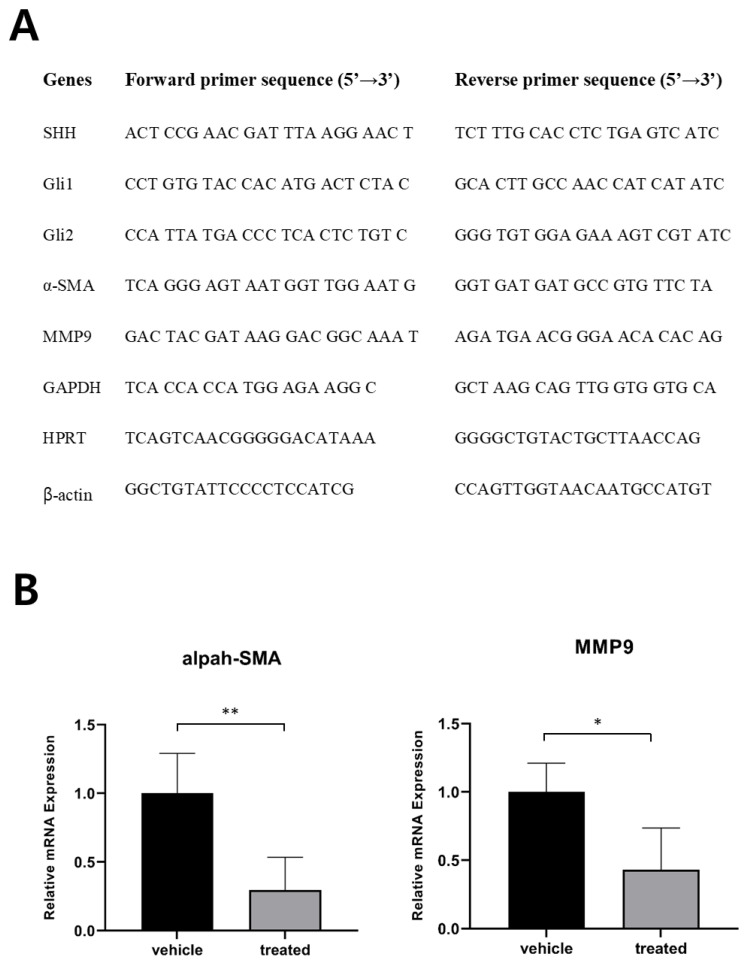
Assessment of gene expression by quantitative RT-PCR. (**A**) primer sequences used for quantitative RT-PCR shown in B and Figure 6D. (**B**) Expression levels of α-SMA and MMP9 in livers of indicated groups (*, *p* < 0.05; **, *p* < 0.01).

From this study, the molecular mechanism underlying how pharmacological suppression of SHH signaling inhibited tumor development in the murine CCC model remains elusive. Of note, it has been reported that hepatic myofibroblasts promote the progression of human cholangiocarcinoma [17,18]. The SHH signaling is an important regulator for differentiation and expansion of myofibroblasts in the liver [19,20]. We investigated levels of myofibroblasts in CCC from mice treated with vismodegib by assessing levels of alpha-smooth muscle actin (α-SMA), a marker for myofibroblasts. Significant reduction of α-SMA levels were found in intrahepatic CCC from mice treated with vismodegib, compared with those from control mice (Figure 7B). Further study is required to precisely determine the role of decreased myofibroblasts in vismodegib-mediated anti-tumor effects on CCC.

In conclusion, our study demonstrates that CCC exhibiting a high activity of SHH signaling is vulnerable to HPI, thus pharmacological inhibition of the SHH signaling pathway is considered an effective molecular target therapy for CCC patients. 

## 4. Materials and Methods

### 4.1. Publicly Available Genomic Data Analyses

Data analysis of transcriptome for patients with CCC was performed using the publicly available database, The Cancer Genome Atlas (accessed on 3 May 2019).

### 4.2. Cell Culture and Treatment

Human intrahepatic CCC cell lines, SNU1079 and SNU-245 were purchased from the Korean Cell Line Bank. The cells were maintained at 37 °C in a humidified atmosphere of 5% CO_2_. Cells were cultured in RPMI1640 with *L*-glutamine (300 mg/L), 25 mM HEPES and 25 mM NaHCO_3_ (Welgene, Gyeongsan, Korea) supplemented with 10% fetal bovine serum (Gibco, Grand Island, NY, USA). Cells were plated 10^5^ cells per well on 6 well plate one day prior to drug treatment. Cells were treated with vismodegib at concentrations of 0, 1, 5, 10, and 20 μM. Cells were harvested at 0, 24, 48, and 72 h after the treatment and then used for cell viability assay (EZ-CYTOX; DoGenBio, Seoul, Korea).

### 4.3. Animal Models

All experiments using mice were approved by the Animal Policy and Welfare Committee of the Yonsei University College of Medicine (Permit Numbers: 2018-0211). Wild-type male C57BL/6 mice were purchased from Orient Bio (Seongnam, Korea). Animals were housed in an animal facility under a 12 h light/dark cycle and were provided food and water ad libitum. Tissues were collected immediately following surgery and stored at −80 °C until processing and use.

### 4.4. Hydrodynamic Transfection and Drug Treatment

The plasmids pT3/EF5a-TAZ^S89A^, pT2/PI3KCA^E545K^, and pPGK-SB13 were described previously [21,22]. Hydrodynamic injection has also been previously described [21]. DNA mixtures of transposons (pT2- or pT3- plasmids) and transposase-encoding vector (pPGKSB13) were suspended in lactated Ringer’s solution and subsequently injected into the lateral tail veins of male 5–6-week-old mice (0.1 mL/g body weight). Mice were randomly assigned to hydrodynamic injection. Drugs were intraperitoneally administered daily beginning 5 weeks after hydrodynamic transfection. Doses of drugs administered was 50 mg/kg/day for vismodegib. All drugs were purchased from Selleckchem. All mice in the control group received an equal volume of 10% DMSO in phosphate-buffered saline (Welgene, Gyeongsan, Korea) by intraperitoneal injection according to the same treatment schedule.

### 4.5. Liver Harvest and Tissue Processing

Mice were deeply anesthetized by intraperitoneal injection of tiletamine/zolazepam (ZoletilTM, 30 mg/kg) and xylazine (10 mg/kg). A midline laparotomy incision was then performed and the maximum possible amount of blood was collected from the inferior vena cava. Pieces of extracted liver were immersed in freshly prepared 10% neutral-buffered formalin and incubated overnight. The remainder of the liver was snap-frozen in liquid nitrogen and stored at −70 °C until subsequent use.

### 4.6. Immunohistochemical Analyses of Mouse Tissue Samples

For immunohistochemistry, paraffin-embedded sections were deparaffinized in xylene and rehydrated in a decreasing graded ethanol series. Antigen epitopes were then unmasked using a 10 mM sodium citrate buffer (pH 6.0) incubation procedure, after which sections were incubated overnight at 4 °C with the primary antibody. After incubation with primary antibody, sections were incubated with the appropriate biotinylated secondary antibody followed by treatment with freshly prepared DAB substrates (Vector Laboratories, Burlingame, CA, USA). Sections were lightly counter-stained with hematoxylin and mounted. The primary antibodies used in the study are anti-YAP/TAZ (Cat# 8418, Cell Signaling Technology, Danvers, MA, USA), anti-Gli2 (ab26056; Abcam, Cambridge, UK), and anti-CK19 (ab133496; Abcam, Cambridge, UK). 

### 4.7. RNA Purification, Reverse Transcription and Real-Time PCR Amplification

Total RNA from extracted livers and cells was collected and purified with an RNeasy Mini Kit (Qiagen, Germany) and converted to cDNA using a Superscript IV Synthesis Kit (Invitrogen, USA). qPCR was performed on a StepOnePlus™ PCR System using PCR master mix (Applied Biosystems, Waltham, MA, USA). The relative expression levels of target genes were normalized to the mean expression levels of three housekeeping genes, *Gapdh*, *Hprt*, and *Actb* (β-actin). All qPCR results were obtained from at least three biological replicates. Primers used for qPCR are shown in Figure 7A.

### 4.8. Protein Etraction and Western Blotting

Liver tissues were homogenized and digested in 1×RIPA buffer containing a protease inhibitor and phosphatase inhibitor cocktail solution (Thermo Scientific, Waltham, MA, USA). Western blotting experiments were performed following a standard protocol. The primary antibodies used were anti-SHH (SC-9024; Santa Cruz Biotechnology, Dallas, TX, USA), anti-Gli1 (ab167388; Abcam, Cambridge, UK), anti-Gli2 (ab26056; Abcam, Cambridge, UK), and anti-GAPDH (# 2118; Cell Signaling Technology, Danvers, MA, USA). 

### 4.9. Statistical Analysis

Statistical analyses were carried out with two-tailed unpaired *t*-tests or Mann Whitney test, where appropriate, using GraphPad Prism Software (GraphPad, La Jolla, CA, USA). All values are expressed as the mean ± SEM. Significant differences between two groups were denoted by asterisks (*, *p* < 0.05; **, *p* < 0.01; ***, *p* < 0.001).

## Data Availability

All the data and materials supporting the conclusions are included in the main paper.
